# Effect of genotype on duodenal expression of nutrient transporter genes in dairy cows

**DOI:** 10.1186/2049-1891-4-49

**Published:** 2013-12-09

**Authors:** Sinéad M Waters, Kate Keogh, Frank Buckley, David A Kenny

**Affiliations:** 1 Teagasc Animal and Bioscience Research Department, Animal and Grassland Research and Innovation Centre, Grange, Dunsany, Co. Meath, Ireland; 2 Teagasc Animal and Bioscience Research Department, Animal and Grassland Research and Innovation Centre, Moorepark, Fermoy, Co. Cork, Ireland

**Keywords:** Bovine, Duodenum, Gene expression, Nutrient transporters

## Abstract

**Background:**

Studies have shown clear differences between dairy breeds in their feed intake and production efficiencies. The duodenum is critical in the coordination of digestion and absorption of nutrients. This study examined gene transcript abundance of important classes of nutrient transporters in the duodenum of non lactating dairy cows of different feed efficiency potential, namely Holstein-Friesian (HF), Jersey (JE) and their F_1_ hybrid. Duodenal epithelial tissue was collected at slaughter and stored at -80°C. Total RNA was extracted from tissue and reverse transcribed to generate cDNA. Gene expression of the following transporters, namely nucleoside; amino acid; sugar; mineral; and lipid transporters was measured using quantitative real-time RT-PCR. Data were statistically analysed using mixed models ANOVA in SAS. Orthogonal contrasts were used to test for potential heterotic effects and spearman correlation coefficients calculated to determine potential associations amongst gene expression values and production efficiency variables.

**Results:**

While there were no direct effects of genotype on expression values for any of the genes examined, there was evidence for a heterotic effect (*P* < 0.05) on *ABCG8*, in the form of increased expression in the F_1_ genotype compared to either of the two parent breeds. Additionally, a tendency for increased expression of the amino acid transporters, *SLC3A1* (*P* = 0.072), *SLC3A2* (*P* = 0.081) and *SLC6A14* (*P* = 0.072) was also evident in the F_1_ genotype. A negative (*P* < 0.05) association was identified between the expression of the glucose transporter gene *SLC5A1* and total lactational milk solids yield, corrected for body weight. Positive correlations (*P* < 0.05) were also observed between the expression values of genes involved in common transporter roles.

**Conclusion:**

This study suggests that differences in the expression of sterol and amino acid transporters in the duodenum could contribute towards the documented differences in feed efficiency between HF, JE and their F_1_ hybrid. Furthermore, positive associations between the expression of genes involved in common transporter roles suggest that these may be co-regulated. The study identifies potential candidates for investigation of genetic variants regulating nutrient transport and absorption in the duodenum in dairy cows, which may be incorporated into future breeding programmes.

## Background

In dairy cow systems feed is the single greatest variable cost, accounting for up to 80% of the costs of production [1]. As profitability is directly linked to the efficient conversion of feed into milk, the identification of feed efficient animals is critically important to the economic sustainability of the enterprise. In dairy cattle, residual milk solids production is a measure of feed efficiency and can be used to identify animals that produce higher amounts of milk solids but have a similar level of feed intake to their herd counterparts [2]. Indeed, studies [3,4] have also shown clear differences between dairy breeds and their feed intake and efficiencies. Schwerin *et al*. [5] reported differences in nutrient utilisation between dairy and beef breeds and, specifically, that the expression of genes involved in nutrient transportation in the liver and intestine differed between Charolais and Holstein bulls. Furthermore, recent data from an Irish study has shown that dairy cow genotype affects the expression profiles of genes involved in energy homeostasis in duodenum and liver [6].

The duodenum plays a critical role in nutrient digestion and absorption and is the site of expression of key signalling molecules regulating energy homeostasis and feed efficiency in cattle [6]. A number of studies have previously examined the effect of diet type on the absorption of nutrients namely, sugar, nucleoside and amino acid in the duodenum of beef cattle [7-9]. However, there is a dearth of information detailing mineral and lipid transporter mRNA abundance in this tissue. Additionally, there is no information available on whether differences exist between contrasting dairy cow genotypes or animals of different feed efficiency potential for the absorption of nutrients in the small intestine. Thus the aim of this study was to determine the effect of dairy cow genotype on the expression profiles of a variety of genes involved in the transportation and absorption of nutrients and minerals in Holstein-Friesian (HF), Jersey (JE) and Holstein-Friesian Jersey cross (F_1_). Gene transcript abundance of five important classes of nutrient transporters, namely nucleoside, amino acid, lipid, sugar and mineral transporters, was investigated.

## Materials and methods

All procedures involving animals were carried out under a licence for the Irish Department of Health and Children in accordance with the European Community Directive 86/609/EC.

### Experimental animals

This study was part of a larger experiment designed to evaluate the performance of three dairy genotypes, HF, JE and F_1_ (JE × HF), on a pasture-based production system. All data were generated at the Ballydague research farm (52°8′N 8°26′W), Teagasc Moorepark Dairy Production Research Centre, Fermoy, Co. Cork, Ireland. Performance data were obtained from 110 animals, representing HF (n = 37), JE (n = 36) and F_1_ (n = 37) cows and was calculated as described by Prendiville *et al*. [3].

A total of 6, 7 and 3 sires were represented in the HF, JE and F_1_, respectively. All F_1_ animals were sired by JE bulls and were born to HF cows. The HF sires were of North American (86%) and New Zealand (14%) origin. The mean predicted transmitting abilities (PTA) (across breed) and standard deviations for the HF sires used were: +163 kg (31.1), +13 kg (7.0), +10 kg (3.0), +0.12% (0.14) and +0.08% (0.06) for milk yield, fat yield, protein yield, fat and protein concentration, respectively (source http://www.ICBF.com, April 2009). Comparable PTAs for the JE sires were: -408 kg (193.5), +8 kg (6.6), -3 kg (7.4), +0.55% (0.27) and +0.24% (0.10). The JE sires were of New Zealand (56%) and Danish (44%) origin. Of the 7 JE sires, 1 was represented in both the JE and F_1_ cows. This sire accounted for 14% and 50% of the JE and F_1_ cows, respectively. All sires were representative of the sires commonly used through AI in Irish dairy herds.

### Tissue sample collection

At the end of lactation, cows were dried off and subsequently fed grass silage *ad libitum* for two months. A sub group of 30 cows from the initial 110 were randomly selected for inclusion in this study representing 10 HF, 10 JE and 10 F_1_. All 30 animals were slaughtered in a licensed abattoir (Dawn Meats, Charleville, Co. Cork, Ireland). Duodenal tissue (5 cm long) was harvested approximately 15 cm distal to the abomasal-duodenal juncture. Tissue samples were washed in DPBS. Epithelial tissue was then scraped from the underlying connective and muscular tissue using a glass microscope slide. The tissue was washed with sterile phosphate buffered saline (PBS), snap frozen in liquid nitrogen and subsequently stored at −80°C. All instruments used for tissue collection were sterilised and treated with RNA Zap (Ambion, Dublin, Ireland) before use.

### RNA extraction and purification

Total RNA was isolated from approximately 40 mg of duodenal epithelial tissue using TRIzol reagent and chloroform (Sigma-Aldrich Ireland, Dublin, Ireland). Tissue samples were homogenised using a tissue lyser (Qiagen, UK), following which the RNA was precipitated using isopropanol. Samples were then treated with RQ1 RNase-free DNase (Promega UK, Southhampton, UK), according to the manufacturers instructions in order to remove any contaminating genomic DNA. The quantity of the RNA isolated was determined by measuring the absorbance at 260 nm using a Nanodrop spectrophotometer ND-1000 (NanoDrop Technologies, DE, USA). RNA quality was assessed on the Agilent Bioanalyser 2100 using the RNA 6000 Nano Lab Chip kit (Agilent Technologies Ireland Ltd., Dublin, Ireland). RNA quality was verified by ensuring all RNA samples had an absorbance (A_260/280_) of between 1.8 and 2. RNA samples with 28S/18S ratios ranging from 1.8 and 2.0 and RNA integrity numbers (RINs), which is a measure of RNA quality based on the integrity of 18 and 28S ribosomal RNA, of between 8 and 10 were deemed high quality.

### Complementary DNA synthesis

Total RNA (1 μg) was reverse transcribed into cDNA using a High Capacity cDNA Reverse Transcription Kit (Applied Biosystems, Foster City, CA,. USA) using the Multiscribe™ reverse transcriptase according to manufacturers instructions. Samples were stored at −20°C for subsequent analyses.

### Primer design and reference gene selection

All the gene specific primers used in this study were designed using the web based software program Primer 3 (http://frodo.wi.mit.edu/primer3/). Potential primers were then subjected to BLAST analysis (http://www.ncbi.nlm.nih.gov/BLAST/), in order to confirm primer specificity and also to ensure that they were homologous to the bovine sequences. All primers for reference and specific target genes were obtained from a commercial supplier (Sigma-Aldrich Ireland, Dublin, Ireland). Details of primer sets used in this study are listed in Additional file 1: Table S1. All amplified PCR products were sequenced to verify their identity (Macrogen Europe, Meibergdreef 39, 1105AZ Amsterdam, The Netherlands).

In order to select stable reference genes relevant to duodenal tissue, analysis of putative reference genes was carried out using the geNorm version 3.4 Excel software package (Microsoft, Redmond, WA). Ct values were transformed to relative quantities using the comparative delta Ct method, to facilitate the calculation of the M value within geNorm software. The software calculates the intra- and intergroup CV and combines both coefficients to give a stability value minus a lower value implying a higher stability in gene expression. A gene was considered to be sufficiently stable within the duodenal tissue, if an M value of less than 1.5 was generated. Within this range of parameters, beta-actin (*ACTB*), glyceraldehydes 3-phosphate dehydrogenase (*GAPDH*) and ribosomal protein SP (*RPS9*) were selected as being suitable reference genes for this study.

### Quantitative real time PCR (qPCR)

Following reverse transcription, cDNA quantity was determined and standardised to the required concentration for qPCR. Triplicate 20 μL reactions were carried out in 96-well optical reaction plates (Applied Biosystems, Warrington, UK), containing 1 μL cDNA (10–50 ng of RNA equivalents), 10 μL Fast SYBR® Green PCR Master Mix (Applied Biosystems, Warrington, UK), 8 μL nuclease-free H_2_O, and 1 μL forward and reverse primers (250–1000 nM per primer). Assays were performed using the ABI 7500 Fast qPCR System (Applied Biosystems, Warrington, UK) with the following cycling parameters: 95°C for 20 s and 40 cycles of 95°C for 3 s, 60°C for 30 s followed by amplicon dissociation (95°C for 15 s, 60°C for 1 min, 95°C for 15 s and 60°C for 15 s). Amplification efficiencies were determined for all candidate and reference genes using the formula E = 10^∧^(−1/slope), with the slope of the linear curve of cycle threshold (Ct) values plotted against the log dilution [10]. Only primers with PCR efficiencies between 90% and 110% were used. The software package GenEx 5.2.1.3 (MultiD Analyses AB, Gothenburg, Sweden) was used for efficiency correction of the raw cycle threshold (**Ct**) values, interplate calibration based on a calibrator sample included on all plates, averaging of replicates, normalization to the reference gene and the calculation of quantities relative to the greatest Ct. Expression of each target gene was normalised to the reference genes and relative differences in gene expression were calculated using the 2^-ΔΔCT^ method [11].

### Statistical analysis

All data were analysed using Statistical Analysis Systems (SAS Institute, Cary, NC; version 9.2). Data were tested for adherence to a normal distribution using the UNIVARIATE procedure of SAS. A Box-Cox transformation analysis was performed using the Transreg procedure in SAS to obtain appropriate lambda values for data which were not normally distributed. These data were then transformed by raising the variable to the power of lambda. A mixed model ANOVA (PROC MIXED, SAS) was conducted to determine the effect of genotype on the relative expression of each gene measured. The Tukey critical difference test was performed to determine the existence of statistical difference between the treatment groups. In an effort to determine whether there was any evidence for potential heterotic effects on the expression of genes of interest, orthogonal contrasts were used to examine differences between the combined mean of expression values for Holstein-Friesian and Jersey animals compared with their F_1_ hybrid. Spearman partial correlation coefficients were calculated to determine associations among gene expression values for each gene in the duodenum in addition to associations amongst gene expression and production efficiency variables, including residual feed intake (RFI), total milk solid (kg) produced over a 305 day lactation period per 100 kg (SOLIDS_WGT), and milk solids produced (kg) per kg of total dry matter intake (SOLIDS_TDMI), using the CORR procedure of SAS. Data were corrected for the fixed effects of both cow genotype and parity.

## Results

### Effect of genotype on cow production efficiency

A more comprehensive explanation of the genotypes, experimental design, grazing management, sward composition, feed intake and production efficiency measurements has been reported [3]. In brief, genotype had a number of statistically significant effects on cow productive efficiency. For example, daily milk solids yield (MLKS; fat and protein yield) was similar for HF and JE but JE was lower than the F_1_ cows (1.33 kg for HF, 1.28 kg for JE and 1.41 for F_1_). Body weight was higher for HF (577 Kg compared to 435 kg for JE with the F_1_ intermediate (520 kg; *P* < 0.05), whereas body condition score was highest (P < 0.05) for the F_1_ cows (3.00 compared to 2.76 for HF and 2.93 for JE).

Dry matter intake (DMI) per unit body weight (3.99 kg for JE compared to 3.39 kg for HF and 3.63 kg for F_1_) and gross production efficiency (0.088 kg for JE compared to 0.087 Kg for F_1_ and 0.079 Kg for HF) was highest in JE. Production efficiency, expressed as net energy intake per MLKS was highest for the F_1_ cows (8.32 UFL compared to 8.11 UFL for HF and 7.45 UFL for JE). Animals were slaughtered at an average of 2.7 (s.d. 0.90), 2.4 (s.d. 0.52) and 2.8 (s.d. 0.46) lactations for HF, JE and F_1_ respectively. In addition, at slaughter Jersey tissues internal organs (or components of the GIT) weighed less than tissues recovered from cows of the other two breed types with the exception of the omasum, which did not differ in size between breeds. On a proportion of metabolic liveweight basis, HF cows had a smaller rumen-reticulum, abomasum and total GIT than both J and F_1_ cows. However, when expressed as a proportion of metabolic liveweight, the weight of these organs did not differ between the three breed types and were similar [12].

### Effect of cow genotype on the expression of genes in duodenal tissue

The effect of cow genotype on the expression of genes involved in nutrient and mineral absorption in the duodenum is presented in Table 1. Out of 27 genes tested, 19 were found to be expressed in duodenal tissue. Of all the genes studied, only one *ABCG8* (*P* = 0.042), was identified as significantly differentially expressed between groups. However, there was a strong tendancy towards mRNA expression levels for *SLC3A1* (*P* = 0.072), *SLC3A2* (*P* = 0.081) and *SLC6A14* (*P* = 0.072) being different between the three genotypes. There was evidence for a heterotic effect (*P* < 0.05) on the duodenal expression of the lipid transporter *ABCG8*, with expression levels higher for the F_1_ genotype, compared with the mean of the two parent breeds. There was no evidence for any potential heterotic effects (*P* > 0.10) for the expression of any other gene studied in duodenal tissue.

**Table 1:** Effect of genotype and potential heterotic effects on the expression of nutrient and mineral transporter genes in the duodenal epithelium of dairy cows

	**Genotype**				**Significance (P-value)**	
**Gene name**	**HF**	**J**	**F**_**1**_	**SEM**^**1**^	**Genotype**	**HF + J vs. F**_**1**_
***Nucleoside transporters***						
*SLC28A1*	4.39	4.23	4.50	0.55	0.95	0.79
*SLC28A2*	4.49	4.48	4.49	0.62	0.86	0.59
*SLC28A3*	4.62	4.76	5.02	0.48	0.84	0.59
*SLC29A1*	0.85	0.87	0.86	0.03	0.95	0.99
***Amino acid transporters***						
*SLC3A1*	3.08	3.48	4.35	0.44	0.15	0.07
*SLC3A2*	0.98	1.11	1.28	0.10	0.13	0.08
*SLC6A14*	3.08	3.48	4.35	0.44	0.15	0.07
*SLC7A1*	1.96	2.04	2.69	0.45	0.48	0.24
*SLC7A6*	4.43	3.45	4.87	0.63	0.29	0.26
*SLC7A7*	0.95	0.96	1.17	0.15	0.54	0.27
*SLC15A1*	2.10	1.98	2.15	0.12	0.59	0.49
***Sugar transporters***						
*SLC2A2*	6.77	6.09	6.72	0.93	0.83	0.81
*SLC2A5*	2.76	2.35	2.97	0.55	0.73	0.57
*SLC5A1*	4.75	3.82	4.74	0.47	0.29	0.46
***Lipid transporters***						
*ABCG8*	4.81^a^	4.66^a^	8.17^b^	1.24	0.04*	0.02*
***Mineral transporters***						
*SLC11A2*	2.82	2.16	2.60	0.08	0.69	0.93
*SLC31A1*	2.48	2.06	3.59	1.05	0.61	0.34
*SLC39A4*	2.51	2.69	4.17	1.12	0.22	0.10
*TRPV6*	4.02	3.91	4.57	0.09	0.92	0.68

HF = Holstein-Friesian; Je = Jersey; F_1_ = Holstein-Friesian x Jersey. SEM^1^ = pooled standard error, Genotype, *P < 0.05. Gene expression values are presented as ratios of cycle threshold (Ct) value for each gene normalized to that of the reference gene after adjustment for efficiencies and interplate variation.

^a,b^Means sharing the same superscript are not significantly different at P < 0.05.

Bold represents significant (P<0.05) results in terms of difference between means in Table 1 or correlations in Tables 2 and 3.

### Associations between gene expression values within duodenal tissue samples

The results of a Spearman correlation analysis which was conducted to examine the associations between gene expression values in the duodenum is presented in Table 2. Gene expression of *SLC28A1* was positively associated with *SLC28A2* (r = 0.89; *P* < 0.05), *SLC3A2* (r = 0.96; *P* < 0.01), *SLC6A14* (r = 0.95; *P* < 0.05) and *SLC2A2* (r = 0.97; *P* < 0.01). Expression values of *SLC28A2* were positively correlated with *SLC3A2* (r = 0.92; *P* < 0.05), *SLC15A1* (r = 0.94; *P* < 0.05), *SLC2A2* (r = 0.97; *P* < 0.01) and *SLC2A5* (r = 0.92; *P* < 0.05). *SLC28A3* was positively associated with *SLC3A1* (r = 0.93; *P* < 0.05), *SLC31A1* (r = 0.90; *P* < 0.01) and *SLC39A4* (r = 0.98; *P* < 0.01). *SLC29A1* was positively associated with *SLC11A2* (r =0.94; *P* < 0.05). *SLC3A1* was positively correlated with *SLC39A4* (r = 0.91; *P* < 0.05). *SLC3A2* was positively associated with *SLC7A6* (r = 0.93; *P* < 0.05), *SLC15A1* (r = 0.93: *P* < 0.05), *SLC2A2* (r = 0.98; *P* < 0.01), *SLC2A5* (r = 0.89; *P* < 0.05), and *SLC31A1* (r = 0.91; *P* < 0.05). *SLC6A14* was positively correlated with *SLC2A2* (r = 0.86; *P* < 0.05). *SLC7A1* was negatively associated with *SLC7A7* (r = −0.95; *P* < 0.05) and positively associated with *ABCG8* (r = 0.98; *P* < 0.01). *SLC7A6* was positively associated with *SLC15A1* (r = 1.0; *P* < 0.001), *SLC2A2* (r = 0.93; *P* < 0.05), and *SLC2A5* (r = 0.94; *P* < 0.05). *SLC7A7* was negatively associated with *ABCG8* (r = −0.88; *P* < 0.05). *SLC15A1* was positively correlated with *SLC2A2* (r = 0.93; *P* < 0.05), and *SLC2A5* (r = 0.94; *P* < 0.05). *SLC2A2* was positively associated with *SLC2A5* (r = 0.88; *P* < 0.05). *SLC31A1* was positively associated with *SLC39A4* (r = 0.96; *P* < 0.01).

**Table 2:** Spearman partial correlation coefficients for the association between the expression of duodenal genes involved in common nutrient transport function

**Nucleoside transporters**					**Amino acid transporters**						**Sugar transporters**			**Mineral transporters**				**Lipid transporter**
***SLC***	***28A2***	***28A3***	***29A1***	***3A1***	***3A2***	***6A14***	***7A1***	***7A6***	***7A7***	***15A1***	***2A2***	***2A5***	***5A1***	***11A2***	***31A1***	***39A4***	***TRPV6***	***ABCG8***
*28A1*	0.89*	0.62	0.62	0.70	0.96**	0.95*	0.28	0.84	−0.01	0.84	0.97**	0.78	0.36	0.75	0.84	0.71	0.83	0.45
*28A2*		0.35	0.75	0.34	0.92*	0.73	0.36	0.94	−0.12	0.94*	0.97**	0.92*	0.49	0.73	0.70	0.49	0.83	0.52
*28A3*			0.21	0.93*	0.68	0.63	−0.24	0.51	0.47	0.51	0.54	0.46	0.42	0.44	0.90*	0.98**	0.61	−0.09
*29A1*				0.14	0.67	0.40	0.77	0.64	−0.58	−0.58	0.70	0.83	0.05	0.94*	0.40	0.26	0.34	0.86
*3A1*					0.66	0.79	−0.16	0.43	0.38	0.43	0.56	0.33	0.23	0.42	0.85	0.91*	0.57	−0.02
*3A2*						0.85	0.20	0.93*	0.08	0.93*	0.98**	0.89*	0.53	0.75	0.91*	0.78	0.89	0.38
*6A14*							0.17	0.67	0.05	0.67	0.86*	0.55	0.22	0.60	0.79	0.69	0.74	0.33
*7A1*								0.10	−0.95*	0.10	0.31	0.33	−0.54	0.73	−0.13	−0.24	−0.18	0.98**
*7A6*									0.15	1.0***	0.93*	0.94*	0.72	0.62	0.82	0.65	0.93	0.28
*7A7*										0.15	−0.04	−0.08	0.69	−0.50	0.41	0.50	0.43	−0.88*
*15A1*											0.93*	0.94*	0.72	0.62	0.82	0.65	0.93	0.28
*2A2*												0.88*	0.46	0.76	0.82	0.65	0.86	0.48
*2A5*													0.57	0.79	0.73	0.57	0.77	0.49
*5A1*														−0.01	0.63	0.54	0.80	−0.40
*11A2*															0.56	0.46	0.39	0.83
*31A1*																0.96**	0.88	0.04
*39A4*																	0.74	−0.08
*TRPV6*																		−0.01

The probability of a coefficient not being statistically different from zero is denoted as follows: *P < 0.05, **P < 0.01 and ***p < 0.001.

Bold represents significant (P<0.05) results in terms of difference between means in Table 1 or correlations in Tables 2 and 3.

### Associations between duodenal gene expression values and animal production efficiency variables

A Spearman partial correlation analysis was conducted to determine the association between the expression of genes involved in nutrient and mineral absorption in the duodenum and feed efficiency variables, previously reported by Prendiville *et al*. [3,13]. Correlation coefficients for these associations are presented in Table 3. Only one association was identified as reaching statistical significance, *viz.* the correlation between *SLC5A1* gene expression and total milk solids produced over a 305 day lactation period (kg) per 100 kg of body weight (r = 0.93; *P* < 0.05).

**Table 3:** Correlation of expression of genes involved in nutrient transporters in the duodenum and production efficiency variables

**Traits**	**RFI**	**SOLIDS_WGT**	**SOLIDS_TDMI**
***Nucleoside transporters***			
*SLC28A1*	−0.33	−0.27	0.31
*SLC28A2*	−0.51	−0.55	0.04
*SLC28A3*	0.05	−0.15	0.20
*SLC29A1*	0.10	−0.23	−0.49
***Amino acid transporters***			
*SLC3A1*	0.04	0.07	0.46
*SLC3A2*	−0.36	−0.46	0.14
*SLC6A14*	−0.28	−0.05	0.55
*SLC7A1*	0.38	0.31	−0.41
*SLC7A6*	−0.57	−0.72	0.02
*SLC7A7*	−0.45	−0.43	0.42
*SLC15A1*	−0.57	−0.72	0.02
***Sugar transporters***			
*SLC2A2*	−0.42	−0.43	0.17
*SLC2A5*	−0.33	−0.64	−0.26
*SLC5A1*	−0.73	−0.93*	0.06
***Lipid transporters***			
*ABCG8*	0.29	0.20	−0.36
***Mineral transporters***			
*SLC11A2*	0.20	−0.05	−0.29
*SLC31A1*	−0.29	−0.43	0.23
*SLC39A4*	−0.10	−0.29	0.23
*TRPV6*	−0.69	−0.69	0.32

The probability of a coefficient not being statistically different from zero is denoted as follows: *P < 0.05, **P < 0.01 and ***p < 0.001.

RFI; Residual Feed Intake.

SOLIDS_WGT; Total milk solids produced over a 305 day lactation period (kg) per 100 kg body weight (kg).

SOLIDS_TDMI; Total milk solid produced over a 305 day lactation period (kg) total dry matter intake.

Bold represents significant (P<0.05) results in terms of difference between means in Table 1 or correlations in Tables 2 and 3.

## Discussion

Heterosis, or hybrid vigour, where progeny show increased fitness relative to their parents [14] is of economic importance in livestock production [15]. Positive effects of heterosis on growth and BW traits [16,17] and feed efficiency [17] have been reported for beef cattle. In dairy cattle, crossbreeding programmes utilising divergent cow breeds such as HF and JE cows have been explored to address the demands of the dairy industry [18]. Differences in feed intake capacity and production efficiency in lactating HF, JE and their F_1_ have previously been presented by Prendiville *et al*. [3]. The resulting F_1_ progeny have demonstrated promise in improving several traits associated with milk production including feed efficiency [3]. Gozho and Mutsvangwa [19] showed that improved production performance with corn and barley diets appeared to be due to greater nutrient absorption in dairy cows fed oats and grass silage diets. It has been postulated that improvements in digestion or absorption of dietary energy and protein are a possible mechanism to explain variation in feed efficiency [20,21]. In the current study we hypothesised that the improvement in feed efficiency observed in the F_1_ genotype is due to an enhancement in nutrient absorption in the GIT possibly mediated through a modification of gene expression in the nutrient transporters.

We have recently shown that key genes involved in energy homeostasis and appetite behaviour, including *POMC* and *GLP1R,* were differentially expressed in the duodenum and liver, between contrasting cow genotypes, in a tissue dependent fashion [6]. There is, however, a dearth of information regarding the effect of dairy cow genotype on the expression of genes involved in nutrient absorption and transport in the small intestine of cattle and their relationship with production efficiency variables. To uncover potential molecular mechanisms controlling the documented differences in production efficiencies between contrasting breeds, an investigation into the expression of nutrient transporter genes was employed. The duodenum, which is the first section of the small intestine, is a major site of nutrient absorption in all animals [22] and has also been shown to be sensitive to nutritional changes [9]. The current study focussed on examining duodenal gene expression profiles. To our knowledge, this is the first examination of the expression of nutrient transporters in the duodenal tissue of dairy cows.

Of all the nutrient transporter genes analysed, the lipid transporter *ABCG8,* was the only gene found to be differentially expressed across genotype. In addition, heterotic effects in the duodenal expression of *ABCG8* were also observed with mean expression higher in the F_1_ animals compared with the mean of the two parent breeds. ABCG8 is a transporter of dietary cholesterol. While it is usually found co-expressed with *ABCG5*, there was no evidence for expression of this latter gene in duodenal tissue of dairy cows in the current study. Viturro *et al*. [23] examined the gene expression of sterol transporters *ABCG5* and *ABCG8* in a range of bovine tissues including the intestine. While expression was detected in the abomasum, jejunum and colon, the duodenum was not examined in that study. We have therefore shown expression of the *ABCG8* gene for the first time in the duodenum of the bovine. The protein encoded by this gene functions to exclude non-cholesterol sterol entry at the intestinal level, promote excretion of cholesterol and sterols into bile, and to facilitate transport of sterols back into the intestinal lumen. It is expressed in a tissue-specific manner in the liver, intestine, and gallbladder. As plant sterols are a major component of the ruminant diet [23] expression of this gene in the duodenum is not surprising. Therefore it is hypothesised that increased expression of this gene in the F_1_ genotype may lead to enhanced transport of plant sterols, potentially lowering serum and milk cholesterol levels and contribute to improved feed efficiency compared to the parent breeds. Future studies should focus on the functional role of ABCG8 in the digestive tract of ruminants and how it may improve feed digestion and nutrient utilisation in cattle. The fatty acid transporter CD36 is frequently detected in tissues such as adipose [24] and mammary [25]. Recently, the expression of CD36 was shown to be dependent on diet and region of the intestine, with greater expression recorded in the upper jejunum compared to the ileum [26] however in the current study mRNA expression of this gene was not detected.

Amino acids are essential for optimal growth in cattle however there is little information available on amino acid transporter proteins expressed by the duodenum in dairy cattle. We observed a strong tendency towards increased expression of the amino acid transporter genes *SLC3A1, SLC3A2* and *SLC6A14* in the F_1_ genotype, compared with the two parent breeds, consistent with the enhanced production efficiency reported for this genotype. SLC3A1 is involved in sodium independent transport of cystine, neutral and dibasic amino acids across the cell membrane. There is little published data available on this gene for cattle but it has been extensively studied in the human [27]. In the current study, expression of *SLC3A1* was strongly correlated with *SLC6A14*, *SLC7A6* and *SLC7A7* mRNA abundance. SLC7A7 is involved in the sodium dependent uptake of certain neutral amino acids and the sodium independent uptake of dibasic amino acids. It requires the co-expression of SLC3A2 to mediate the uptake of arginine, leucine and glutamine which probably explains the high correlation between these two genes. SLC3A2 is involved in light chain amino acid transport and functions as a sodium independent transporter of large neutral amino acids such as leucine, arginine, tyrosine and phenylalanine. SLC6A14 has a role involved in the sodium and chloride dependent transportation of neutral and basic amino acids. In a study by Liao *et al*. [8] the regulation of this gene amongst others was shown to be strongly regulated by diet. Furthermore gene expression of *SLC5A1* was negatively correlated with total milk solids produced over a 305 day lactation period (kg) per 100 kg of body weight. Liao *et al*. [8] found expression of SLC7A9 to be extremely low in the duodenum of beef steers and indeed, in our study, no expression of this gene was detected in the duodenal tissue of dairy cows. This could also be due to diet effects as animals in the current study were fed grass only while steers in the study of Liao *et al*. [8] were fed cornstarch, partially hydrolyzed by a heat-stable α-amylase. Chen *et al.*[28] found the gene *SLC15A1* to be expressed in the duodenum, jejunum and ileum of cattle while there was no expression detected in stomach, large intestine, liver, kidney and longissimus muscle tissue indicating that this gene is only expressed in the GIT.

In cattle, microbial-derived nucleic acids serve as a source of N and are absorbed as nucleosides through the small intestinal epithelia. Nucleosides are important nutrients for the development of gut and immune system function [29]. A supply of nucleosides is essential for many biological processes during animal development and growth, including DNA and RNA syntheses, energy (ATP) production, N and P recycling, cell signalling, and modulation of gene expression [29]. Liao *et al*. [30] showed that mRNA for nucleoside transporters are expressed throughout the small intestinal epithelia of growing beef steers and can be increased by augmenting the luminal supply of nucleotides. Nucleoside carriers bind to sodium ions as well as the nucleosides being transported. Consistent with our study, Liao *et al*. [30] found that *SLC28A1*, *SLC28A2*, *SLC28A3*, *SLC29A1* were expressed in the duodenum of beef steers. However their group also detected mRNA expression of SLC29A2 which we failed to detect in dairy cow duodenal tissue in our study potentially due to differences in the basal diet offered. SLC28A1 is a sodium coupled nucleoside transporter which has a higher affinity for binding to pyrimidines such as cytosine and thymine which enters a cell across a concentration gradient and uses the flow of sodium ions for transport into cells [31]. The expression of this gene was highly correlated with the expression of *SLC28A2*, which also codes for a sodium coupled nucleoside transporter and functions in the same manner. The high level of correlation could be due to the fact that SLC28A2 has a high affinity for purines such as adenosine and guanine and the expression of both of these genes are required for equal absorption of purines and pyrimidines. SLC28A3 is both purine and pyrimidine selective and functions in a similar fashion to both SLC28A1 and SLC28A2. Expression of *SLC28A3* is highly correlated with *SLC29A1* which is an equilibrative transporter. Unlike the other three nucleoside transporters studied, SLC29A1 is sodium independent and mediates the influx and efflux of nucleosides across a cell membrane. While there are studies published on the expression of this gene in cattle intestines [30], it has been extensively studied in humans due to its potential in aiding the uptake of chemotherapeutic drugs. None of the nucleoside transporters examined in the current study were differentially expressed between genotypes.

The absorption of monosaccharides from the small intestinal lumen of cattle involves sugar transporters, such as sodium-dependent glucose transporter 1 (encoded by the gene *SLC5A1*) which transports glucose and galactose; whereas glucose transporter (GLUT) 5 (GLUT5; encoded by the gene *SLC2A5*) transports fructose, across the apical membrane of enterocytes. Liao *et al*. [8] examined the expression profiles of glucose transporters along the intestinal tract. SLC5A1 is a sodium-glucose co-transporter and transcription of this gene has been extensively studied in humans. Work on the bovine *SLC5A1* gene has been conducted by Wood *et al*. [32] and Liao *et al*. [8]. Recently the expression of SLC5A1 in small intestinal epithelia was found to be influenced by the level of milk replacer fed to bull calves [26] and suggests that feeding high levels of milk replacer to calves can offer an advantage for greater uptake of lactose. SLC2A2 is a facilitated glucose transporter and is highly conserved among mammals such as humans, dogs, mice and rats. SLC2A5 is a cytochalasin B (a mycotoxin) sensitive fructose transporter. In our study expression of *SLC2A5* was highly correlated with that of *SLC2A2*, possibly due to the fact that they both transport sugars. While we failed to detect an effect of genotype on the expression of sugar transporter genes here, a negative association was observed between the expression of the glucose transporter gene SLC5A1 and total lactational milk solids corrected for body weight. Expression levels of SLC5A1, SLC2A2 and SLC2A5 were highly correlated in the current study. Similar to amino acid transporters, the expression of the sugar transporters is possibly co-regulated.

## Conclusions

Taken together with the associated study of Alam *et al*. [6]. These data suggest a possible role for DEG in enhancing feed and production efficiency of dairy cows through improved facilitated absorptive capacity in the duodenum. There is evidence of enhanced expression of key genes involved nutrient transport in the F_1_ genotype, compared with the two parent breeds, consistent with the enhanced production efficiency reported for this genotype. Expression of some genes involved in common nutrient transport roles are positively correlated, suggesting that these may be co-regulated. However a global gene expression approach, using tools such as microarrays or RNAseq, across regions of the GIT between breeds and individuals within breeds, is required to gain a greater understanding of the molecular control of feed efficiency and the contribution of GIT tissues in dairy cows. Furthermore, this study identifies potential candidates for investigation of genetic variants regulating nutrient transport and absorption in the duodenum in dairy cows, which may be incorporated into future breeding programmes.

## Abbreviations

HF: Holstein-Friesian; JE: Jersey; cDNA: Complementary DNA; RT-PCR: Real time polymerase chain reaction; ANOVA: Analysis of variance; SAS: Statistical analysis systems; PTA: Predicted transmitting abilities; PBS: Phosphate buffered saline; Ct: Cycle threshold; DMI: Dry matter intake; GIT: Gastrointestinal tract; BW: Body weight; ME: Metabolisable energy; mRNA: Messenger RNA; N: Nitrogen; ATP: Adenosine tri-phosphate; P: Phosphate; DEG: Differentially expressed genes.

## Competing interests

The authors declare that they have no competing interests.

## Authors’ contributions

SW and DK conceptualised the study and were responsible for experimental design. FB performed the animal study and collected data relating to animal performance. FB and DK collected the duodenal tissue. KK and SW performed real time PCR analysis of duodenal tissue. DK performed statistical analysis. SW, DK and KK participated in the data collection, data analysis and interpretation and drafted the manuscript. All authors approved the final version of the manuscript for publication.

## Supplementary Material

Additional file 1: Table S1 Bovine oligonucleotide primers used for qPCR. Click here for file
