# Supplementary material for: Effect of genotype on duodenal expression of nutrient transporter genes in dairy cows
Source: J Anim Sci Biotechnol. 2013 Dec 9;4(1):49. doi: 10.1186/2049-1891-4-49 (PMC3881024; doi:10.1186/2049-1891-4-49)
Supplement: Additional file 1: Table S1 — Bovine oligonucleotide primers used for qPCR. [file 2049-1891-4-49-S1.doc]

**Additional file 1**

**Table S**1. Bovine oligonucleotide primers used for qPCR

| **Gene**  **Symbol** | **Primers (5’ to 3’)** | **Product size** | **Efficiency^1^** | | **Accession**  **Number** |
| --- | --- | --- | --- | --- | --- |
| ***Nucleoside transporters*** | | | | | |
| *SLC28A1* | F: AGAAGTGAGGAAGGCGTGAA  R: TTGATGAAGTCCAGCACAGC | 146 | 0.91 | | NM_001037458 |
| *SLC28A2* | F: GGATTTGCCAATCTTAGTTCCCA  R: GTCACTCTTCCGGTGGGGTA | 75 | 1.02 | | EF469827 |
| *SLC28A3* | F: GAACCTGGATTTATGGCTTTTG  R: TGAAGCACCAGCATCACTGT | 78 | 0.98 | | EF469828 |
| *SLC29A1* | F: CCTCCGTGGCCATGATCT  R: CGAAGGCACTTTCCGACA | 60 | 1.09 | | EF469829 |
| *SLC29A2* | F: ACCTGGAGTGCTGCTAGGAA  R: CCTCTTTTCCCTGATGACCA | 124 | ND^2^ | | NM_00103269 |
| *SLC29A3* | F: CTGATGGCAAGATGGGAAGT  R: TAGTGGAAAGTGGGGTTTGC | 129 | ND | | NM_001080223 |
| *SLC29A4* | F: CCAGGAACCTCGATTGTGTT  R: CCCCAGGGCTAAGAGGTAAC | 138 | ND | | NM_001193196 |
| ***Amino acid transporters*** | | | | | |
| *SLC3A1* | F: CAAAGGGTGTTGATGGCTTT  R: GCAGCTGCGAGTAGTGAGTG | 126 | 0.93 | | NM_001034633 |
| *SLC3A2* | F: ACCCCAGTGTTCAGCTATGG  R: ATGCTGAGGTGTTGGGAAAG | 118 | 0.99 | | NM_001024488 |
| *SLC6A14* | F: GTTGGGCTGATGGAAACTGT  R: CCGTTGGAGTGCCACTTTAT | 194 | 1.09 | | NM_001098461 |
| *SLC7A1* | F: TCAACCAGCCTCCTAGCACT  R: AGGCAGAGCCCATGAGTAGA | 115 | 1.04 | | NM_001135792 |
| *SLC7A6* | F: TGTTCTGCATTTGCTCCTTG  R: AAAGCAAAGCTTCTGGGTGA | 197 | 1.06 | | NM_001075937 |
| *SLC7A7* | F: CTCTCCATTGGCATCTCCAT  R: AAGTCACAGCAACAGCATCG | 118 | 1.02 | | NM_001075151 |
| *SLC7A9* | F: TGCAGCCTCCTAGAACTGGT  R: GCTCCTTTTGGAGACTCGTG | 119 | ND | | NM_001035054 |
| *SLC15A1* | F: TCGAAGACATCTCCCCAAAC  R: ACCGACTTCATGTTGGAAGG | 139 | 1.03 | | NM_001099378 |
| ***Sugar transporters*** | | | | | |
| *SLC2A2* | F: AGCTATGGCACATCCTGCTT  R: CAAGCTTTTCTTTGCCTTGG | 134 | 0.98 | | NM_001103222 |
| *SLC2A5* | F: ACCTTGCTGTGGTCGGTTAC  R: TTAAGAGTGCAGGCACGATG | 145 | 0.98 | | NM_001101042 |
| *SLC5A1* | F: CCCAAAATGACGAAGGAAGA  R: TCAGCAGAGAGCAGGACTCA | 161 | 1.04 | | NM_174606 |
| *SLC5A10* | F: CCAGCAGGAACACAGTGAGA  R: CCAGCAGCACATAGGTAGCA | 183 | ND | | NM_001001442 |
| *Glut 4* | F: ACCTTATGGCCACTCCTCCT  R: CTCAGCCAACACCTCAGACA | 180 | ND | | NM_174604 |
| ***Lipid transporters*** | | | | | |
| *ABCG5* | F: AGCTCAGGCTCAGGGAAAAC  R: TCGCTCTGCAGGACGTAG | 188 | ND | | NM_001024547 |
| *ABCG8* | F: GCATTGACAGACGAAGCAAA  R: CACTCTGCTCCCTTGACTCC | 132 | 1.07 | | NM_001024663 |
| *CD36* | F: AGCAGGTGCTGAACCTCAGT  R: CTGGCATTAGAATCCCTCCA | 124 | ND | | NM_174010 |
| ***Mineral transporters*** | | | | | |
| *SLC11A2* | F: TGTCTCGGTGTTTGCTGAAG  R: AAAGGTGAGTGTGGGGACTG | 92 | 1.09 | | NM_001101103 |
| *SLC31A1* | F: GTTGGCCTCTGTTGTGGTTT  R: CAAGGGGTTGGAATGAGAGA | 184 | 1.07 | | NM_001100381 |
| *SLC39A4* | F: CTCCTGGTCCTGCTTGAGAG  R: ACACAGGCCTGCTGAGAAGT | 110 | 1.06 | | NM_001046067 |
| *TRPV6* | F: GCACCTTTGAGCTGTTCCTC  R: GCAGCGTAGGTGATGCTGTA | 88 | 1.03 | | NM_001206189 |
| ***Reference genes*** | | | | | |
| *ACTB* | F: AGCAAGCAGGAGTACGATGAGT  R: ATCCAACCGATGCTGTCA | 147 | | 0.98 | NM_173979.3 |
| *RPS9* | F: CCCTCCACGATGCCAAAGT | 64 | | 1.04 | NM_001101152 |
|  | R: CCTCCAGACCTCACGTTTGTTC |  | |  |  |
| *GAPDH* | F: GGCGTGAACCACGAGAAGTATAA  R: CCCTCCACGCTGCCAAAGT | 119 | | 0.98 | NM_001034034 |

^1^E=10^ (−1/slope), ^2^ND = Not detected
